# A mixed methods study evaluating acceptability of a daily COVID-19 testing regimen with a mobile-app connected, at-home, rapid antigen test: Implications for current and future pandemics

**DOI:** 10.1371/journal.pone.0267766

**Published:** 2022-08-08

**Authors:** Nadia Nguyen, Benjamin Lane, Sangwon Lee, Sharon Lipsky Gorman, Yumeng Wu, Alicia Li, Helen Lu, Noemie Elhadad, Michael Yin, Kathrine Meyers

**Affiliations:** 1 The Aaron Diamond AIDS Research Center, Columbia University, New York, New York, United States of America; 2 Department of Biomedical Engineering, Columbia University, New York, New York, United States of America; 3 Department of Biomedical Informatics, Columbia University, New York, New York, United States of America; 4 Vagelos College of Physicians and Surgeons, Columbia University, New York, New York, United States of America; 5 Department of Medicine, Columbia University, New York, New York, United States of America; U.S. Food and Drug Administration, UNITED STATES

## Abstract

**Background:**

Widespread use of at-home rapid COVID-19 antigen tests has been proposed as an important public health intervention to interrupt chains of transmission. Antigen tests may be preferred over PCR because they provide on-demand results for relatively low cost and can identify people when they are most likely to be infectious, particularly when used daily. Yet the extent to which a frequent antigen testing intervention will result in a positive public health impact for COVID-19 will depend on high acceptability and high adherence to such regimens.

**Methods:**

We conducted a mixed-methods study assessing acceptability of and adherence to a daily at-home mobile-app connected rapid antigen testing regimen among employees of a US-based media company. Acceptability was assessed across seven domains of the Theoretical Framework of Acceptability.

**Results:**

Among 31 study participants, acceptability of the daily testing intervention was generally high, with participants reporting high perceived effectiveness, intervention coherence, and self-efficacy; positive affective attitude; acceptable degree of burden and opportunity cost; and assessing the intervention as ethical. 71% reported a preference to test daily using an at-home antigen test than weekly employment-based PCR. Mean adherence to the 21-day testing regimen was 88% with 43% of participants achieving 100% adherence, 48% testing at least every other day, and 10% testing less than every other day.

**Conclusions:**

Despite overall high acceptability and adherence, we identified three implementation challenges that must be addressed for frequent serial testing for COVID-19 to be implemented at scale and have a positive public health impact. First, users need guidance on how and when to adapt testing frequencies to different epidemiological conditions. Second, users and institutions need guidelines for how to safely store and share test results. Third, implementation of serial testing strategies must prioritize health equity and protect those most vulnerable to COVID-19.

## Introduction

The use of at-home rapid COVID-19 antigen tests has been identified as an important public health intervention because such tests provide on-demand results for relatively low cost and can identify people when they are most likely to be infectious. Although antigen tests are not as sensitive as molecular tests like real-time PCR for detecting COVID-19, this limitation can be overcome by testing more frequently, including as often as daily. There is growing evidence from both modeling [1,2] and real-world studies [3–6] that antigen tests used frequently perform as well, and in some cases better at controlling the spread of COVID-19, than PCR administered less frequently.

**Fig 1:**
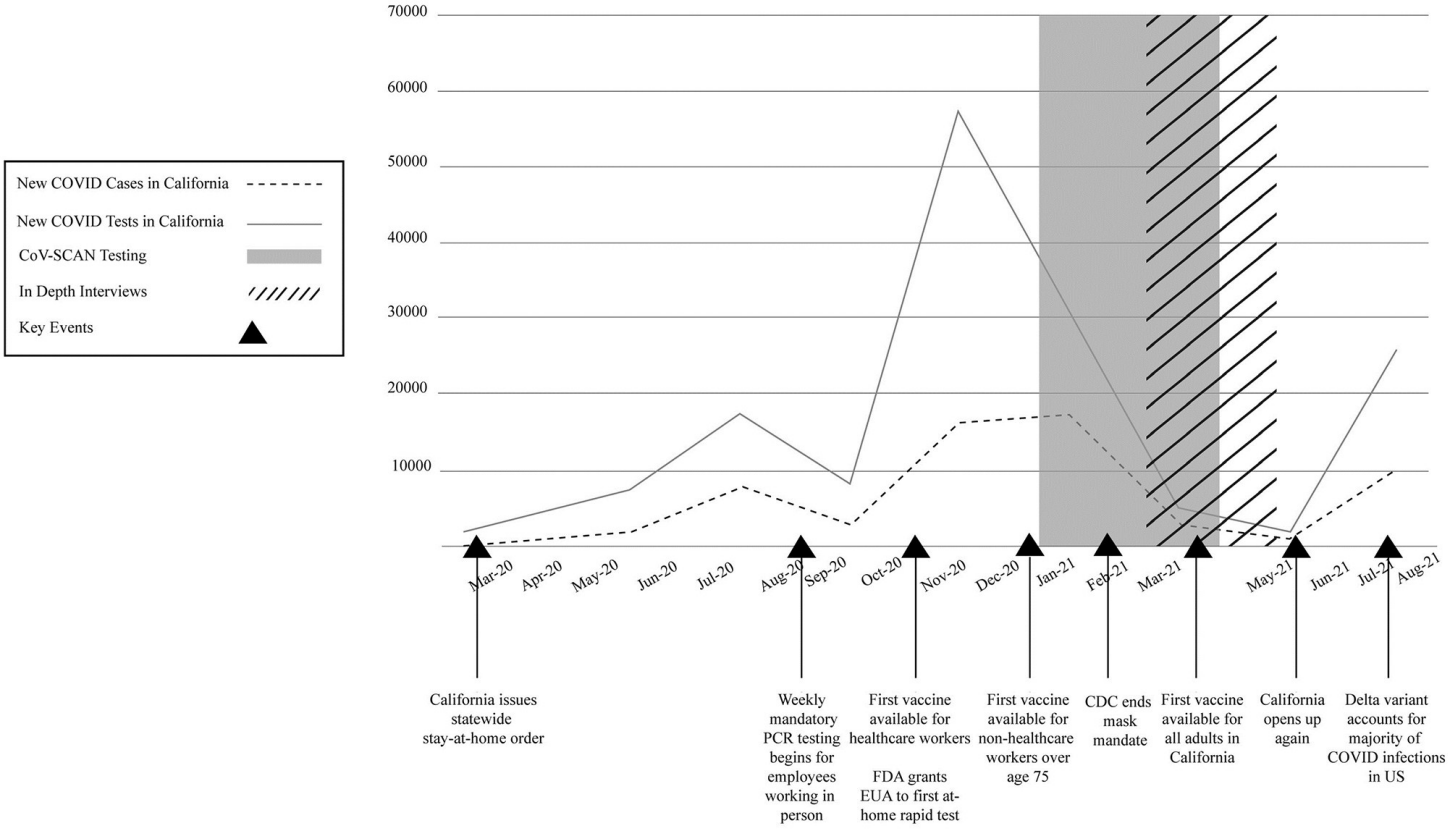
Timeline of key study and COVID-19 pandemic events.

Although antigen tests were developed relatively early in the pandemic, with the first at-home antigen test gaining Food and Drug Administration (FDA) approval for use in the United States (US) in December 2020 (Fig 1), they have been unavailable and underutilized for much of the pandemic in favor of more sensitive but also more expensive and time-consuming lab-based PCR tests [7]. In contrast, at-home antigen testing is a cornerstone in COVID-19 control measures in many European countries, including the UK and Germany, where such tests have been provided for free or at very low cost and have been widely used [7–9]. Recognizing the important role of at-home antigen tests, the Biden administration has taken a number of steps to significantly increase the availability and use of at-home antigen tests, including scaling up production of such tests; investing $2 billion to distribute free tests throughout the community; selling tests at cost; mailing free tests to households; and mandating tests be reimbursed through insurers [10–12]. These steps have the potential to dramatically increase the availability of and access to at-home antigen in the US.

Yet the extent to which a massive influx of at-home antigen tests to the American public will result in a positive public health impact will depend on whether and how the tests are used. To date, there is limited real-world data on the acceptability of at-home antigen testing, particularly when used serially, where they have been shown to be most effective. Previous implementation studies of numerous evidence-based interventions, particularly those requiring sustained participation and adherence, indicate that acceptability is critical to intervention uptake and success [13–15]. Even if at-home antigen tests become widely available, their public health impact will be limited if frequent testing is not acceptable and adopted at scale. To address this knowledge gap, we conducted a mixed-methods study informed by Sekhon’s Theoretical Framework of Acceptability (TFA) [16] to evaluate the acceptability of and adherence to a daily testing regimen among employees of a large media company in the US.

## Methods

### Research design

We conducted a mixed-methods observational study from February-June 2021 to examine factors that influenced the acceptability of and adherence to a serial testing regimen using a self-administered at-home rapid antigen test (“at-home antigen test”) in a workplace setting among employees at two divisions of The Walt Disney Company (“Disney”), a media company based in Southern California in the US. This mixed-methods acceptability study was nested within a larger parent study (N = 93) testing the effectiveness of a daily testing regimen using an at-home antigen-test against weekly testing with PCR at Disney. Participants in the parent study were mailed at-home COVID-19 antigen test kits to their home address and asked to self-test daily for 21 days and, as a condition of their employment, were also concurrently testing at least weekly for COVID-19 using PCR. In our nested acceptability study, after providing written informed consent, participants were asked to complete a one-time quantitative survey (N = 31), and a subset of participants contributed a one-time qualitative in-depth interview (N = 15) at the conclusion of 21 days of daily testing. Participants had a choice to complete both the quantitative survey and qualitative interview or just the survey. Participants who completed the survey were entered into a drawing for a $100 gift card, and participants who completed the interview received a $25 gift card. All study procedures were reviewed and approved by the Columbia University Irving Medical Center’s Institutional Review Board.

### Recruitment and study population

Participants in the acceptability study were informed of the goals of the study and recruited from the parent study via email. To be eligible for the parent study, participants were 18 years of age or older; owned a smartphone; understood and read English; had not been diagnosed with COVID-19 in the past 90 days; had not received any COVID-19 vaccine doses at enrollment; and were willing to share their weekly PCR results. There were no additional inclusion criteria for the acceptability study aside from willingness to complete the quantitative survey and being at least 14 days into the parent. COVID-19 vaccinated employees were excluded because at the time of recruitment, vaccination was believed to provide strong protection against breakthrough infections.

### At-home rapid antigen test and mobile app

Participants used an investigational at-home antigen test that is currently under review with the FDA for Emergency Use Authorization. Test results were available to participants after 15 minutes (for test details, see S1 Fig) The test also included a paired smart phone mobile application (mobile app) that had the following functions: provided step-by-step testing instructions; captured image of test result and automatically interpret result (positive, negative, invalid; securely store test result history; and transmit daily test result to the study team.

## Data collection

### Theoretical framework

The quantitative survey and in-depth interview questions were informed by Sehkon’s Theoretical Framework of Acceptability (TFA) [16]. The TFA defines intervention acceptability as a multi-faceted construct that reflects the extent to which people consider the health care intervention to be appropriate, based on anticipated or experienced cognitive and emotional responses to the intervention. The TFA consists of seven constructs which we adapted to measure the acceptability of the intervention: affective attitude, burden, perceived effectiveness, ethicality, intervention coherence, opportunity costs, and self-efficacy (Table 3).

### Adherence to daily testing regimen

Participants submitted daily test results to the study team by scanning a picture of the test cassette using the mobile app.

### Quantitative survey

Each of the TFA’s seven constructs of acceptability was evaluated by one to three statements which participants rated on a five-point Likert scale (strongly disagree = 1 to strongly agree = 5). As a global measure of acceptability, participants were also asked whether, if given a choice, they would prefer to test for COVID-19 using the current PCR regimen or test daily with an at-home antigen test. We measured sociodemographic factors, including age, race/ethnicity, sex, education, living environment, and financial stability. Participants self-administered the 20-minute survey using REDCap, a secure web platform for administering online surveys.

### In-depth interview

We conducted in-depth interviews with participants to more deeply understand their current and previous COVID-19 testing experiences and the acceptability of the daily COVID-19 testing regimen. The interview guide was designed to assess the seven constructs in the TFA. Interviews were approximately 60 minutes long and conducted over Zoom by a team of four researchers experienced in qualitative interviewing (NN, BL, KM, and YW). Of the four researchers: two identified as Asian cis-gendered females, 1 White cis-gendered female, and 1 White cis-gendered male; all four lived in New York City. All four were employed by Columbia University, which made rapid turnaround PCR testing available as needed in addition to random surveillance testing, and all were also using at-home antigen tests in their personal lives. Interviews were summarized in debrief forms immediately after completing the interviews and were audiorecorded and transcribed by a paid transcription service.

### Data analysis

#### Quantitative analysis

We summarized participant characteristics by estimating mean, standard deviation, frequency and proportion for all categorical and continuous sociodemographic and health factor variables, respectively. Additionally, we estimated the mean, standard deviation, median, and interquartile range (IQR) for each TFA construct measure and also estimated the frequency and proportion of participants who stated that they strongly agreed or agreed with each statement. We calculated overall adherence by taking the number of submitted test results and dividing it by the 21 days of the study. We categorized adherence into three groups and estimated the number and proportion of participants per group: 100% (tested daily), 50%-99% (tested at least every other day), and <50% (tested less than every other day). Finally, we calculated the frequency and proportion of participants who stated they would prefer testing daily with a rapid antigen test versus their current PCR regimen if given the choice.

#### Qualitative analysis

Interview transcripts were analyzed by a team of three researchers in Dedoose using Template Analysis, which is appropriate for studies using a priori themes and exploring perspectives of different groups in an organizational context [17]. We initiated data analysis with a top-down approach, using the seven constructs in the TFA to create an organizational template. One member of the research team created a preliminary codebook by reading through interview debrief forms and identifying subconstructs within each of the TFA constructs. With the seven TFA constructs listed as parent codes and the subconstructs as child codes, the initial codebook was applied to two interview transcripts in Dedoose. A second researcher then applied the preliminary codebook to two more interviews and the codebook was further discussed and refined. All three coders then re-coded the initial four interviews using the finalized codebook to maximize reliability across coders. The three coders then individually coded the remaining 11 interviews, meeting on an ad hoc basis to resolve coding questions, and using memos to note emerging themes. The coders generated code reports for each TFA construct, identified key quotes for each construct and subconstruct, and held team meetings to discuss emergent themes that conceptualized how each construct either directly or indirectly influenced acceptability and adherence. Lastly, we created quote matrices for each construct and subconstruct using the identified key quotes organized by emergent themes.

## Results

### Participant characteristics

Of the 63 eligible Disney employees, we enrolled 31 participants who completed the online survey, of whom 15 also agreed to an in-depth interview between February-June 2021 (Table 1, Fig 1). The 15 interview participants were similar to the overall sample of the survey, with regard to sociodemographic and health factors and adherence.

**Table 1:** Study participant demographics at enrollment (N = 31).

	N	%
Mean age (SD)	44.13	10.44
Race/ethnicity		
Black / African-American / Afro-Caribbean	2	6.45%
Hispanic	5	16.13%
White	19	61.29%
Asian / Pacific Islander	4	12.90%
Native American / Alaskan Native	0	0.00%
Skipped	1	3.23%
Sex		
Male	11	35.48%
Female	20	64.52%
Intersex	0	0.00%
Highest level education achieved		
Less than high school	0	0.00%
High school	0	0.00%
College	17	54.84%
Above college	14	45.16%
Lives with		
Yes, I live alone	8	25.81%
No, I live with family members	21	67.74%
No, I live with my coworkers	2	6.45%
No, I live with other roommates (who are not my family members or coworkers)	0	0.00%
Lives with an at-risk population		
Children under 18	12	38.71%
Someone who is in a COVID-19 risk group (e.g., people over 65 years and/or with chronic disease)	2	6.45%
None of the above	17	54.84%
Any of the above	14	45.16%
Has your household income changed significantly since February 2020?		
Yes, my household income is more	2	6.45%
Yes, my household income is less	11	35.48%
No, my household income is about the same	18	58.06%
In the past month, how difficult has it been for you to cover your expenses and pay all your bills?		
Very difficult	1	3.23%
Somewhat difficult	4	12.90%
Not at all difficult	26	83.87%
Don’t know	0	0.00%
Do you think there would be a negative financial consequence for you if you got COVID-19?		
Yes	20	64.52%
No	10	32.26%
I’m not sure	1	3.23%
It is easy for me to download new apps.		
Strongly disagree	0	0.00%
Disagree	1	3.23%
Neither disagree nor agree	5	16.13%
Agree	12	38.71%
Strongly agree	13	41.94%
It is easy for me to become comfortable using new apps.		
Strongly disagree	0	0.00%
Disagree	0	0.00%
Neither disagree nor agree	4	12.90%
Agree	18	58.06%
Strongly agree	9	29.03%

#### Adherence to daily testing regimen

Mean adherence to the 21-day testing regimen was 88% (SD: 21%, Median 95%, IQR: 90%, 100%) with 43% of participants achieving 100% adherence, 48% achieving 50–99% adherence, and 10% achieving less than 50% adherence (Table 2).

**Table 2:** COVID-19 testing adherence and preference.

	N	%
Adherence to 21 days of COVID-19 testing using at-home antigen test		
100%	13	41.93%
50–99%	15	48.39%
<50%	3	9.68%
If given a choice, would you prefer to test for COVID-19 using current PCR testing regimen or daily antigen testing		
PCR regimen	6	19.35%
Daily antigen test regimen	22	70.97%
Neither, I only want to get tested when I need to	3	9.68%

### Testing preference

Most participants (71%) reported that if given the choice, they would prefer to test for COVID-19 daily using an at-home antigen test than weekly using PCR, while 19% preferred PCR, and 10% preferred only testing when necessary (Table 2).

### Acceptability of the daily testing regimen

#### Affective attitude

Participants reported overall positive feelings about the intervention though only 58% agreed with the statement “I like using the test every day” (see Table 3 for mean and median Likert scale scores). The overwhelming feeling reported by participants was comfort and peace of mind (Table 4, Affective attitude 1). Testing negative gave them needed reassurance to go about their daily lives and participants reported feeling “liberated” to engage in everyday activities without fear of infecting others (Affective attitude 2, 3). Participants also described using the tests after engaging in a “high risk” activity or potentially being exposed to COVID-19 (Affective attitude 3). Notably, a small number of participants described significant anxiety due to living with someone who was immunocompromised; for these participants, the ability to test daily or on demand provided significant mental health benefits (Affective attitude 4).

**Table 3:** Quantitative measurement of Sekhon’s domains of acceptability of a COVID-19 daily antigen testing regimen in an employment context (N = 31).

Domains of Acceptability		Mean	SD	Median	25%, 75%
Affective Attitude	Using the antigen test every day makes me nervous. (R)	1.42	0.56	1	1, 2
	I like using the antigen test every day.	3.71	1.01	4	3, 4.5
Burden	It is burdensome to learn your COVID status on a daily basis.	2.26	1.29	2	1, 3
	It is easy wait 15 minutes every day to find out your results.	3.35	1.23	4	2, 4
Self-Efficacy	It is easy to learn to use the rapid antigen test	4.23	0.88	4	4, 5
	It is easy understand the results of the test.	4.58	0.50	5	4, 5
	I have developed a habit of using the antigen test in my everyday routine.	3.42	1.06	4	3, 4
Opportunity Cost	It is easy for me to find a time to use the antigen test every day.	3.74	1.09	4	3, 4.5
	It is easy for me to find a place to use the antigen test every day.	4.16	0.78	4	4, 5
Coherence	Testing every day using a rapid antigen test to prevent the spread of COVID-19 makes sense to me.	4.16	1.00	4	4, 5
Perceived Effectiveness	I trust the results of the rapid antigen test	4.19	0.91	4	4, 5
	I am confident that testing daily using a rapid antigen test will help keep the people I work with safe from COVID-19.	4.35	0.75	4	4, 5
Ethicality	I believe that it is ethical for an employer to require its employees test regularly for COVID-19.	4.39	0.72	5	4, 5
	I believe testing for COVID-19 should be a personal choice.	2.19	0.95	2	2, 2.5
	I believe sharing COVID-19 test results should be a personal choice.	3.06	1.36	3	2, 4

1 = Strongly disagree, 5 = Strongly agree.

**Table 4:** Acceptability of a COVID-19 daily antigen testing regime in an employment context according to Sekhon’s seven domains of acceptability.

Construct	Quote
**Affective attitude** How a participant felt overall about testing daily.	**Affective attitude 1**: "The fact that the test came to my house, and I got up every morning, and followed the directions, and got results immediately which was very comforting actually” (PID 39, Adherence: 95%). **Affective attitude 2**: "When I think about a test like this, I think about the reassurance, and then I think about leveraging it. Anytime I’m entering any type of higher risk situation, say, I’m going to go to a concert where I know, I’m going to be inside with 1000 people. Do I take one then? […] Or if I’m going to visit my grandmother who’s 95. So, I think the reassurance situations, I could see myself leveraging it. (PID 20, Adherence 95) **Affective attitude 3**: "I felt like socially liberated a little more after these tests. Like, you know, I can meet that person outside. […] There were some instances where like, I would maybe go to the grocery store, somebody would sneeze near me and I would like sprint home and like do these COVID tests to be like, you know, am I going to die?”(PID 25, Adherence 33) **Affective attitude 4**: "I live with my brother who’s immunocompromised […] And I was worried that in between my week, my once-a-week testing appointments at work that I could contract COVID and infect my brother […] So, I personally felt a weight on my shoulders to be more responsible. And I, I loved that this test allowed me the idea of that where I could actually have the ability to test daily to make sure that my roam to Whole Foods won’t kill my brother. You know, just things like that that really worked wonders for just my peace of mind." (PID 25, Adherence 33)
**Burden** The amount of effort a participant perceived was required to test daily	**Burden 1**: “With [the at-home test] it took me 18 minutes, even with a really convenient local site, that saves me 40 minutes, getting the car, and you’re putting on pants.” (PID 21, Adherence: 80%) **Burden 2**: "You’re able to do it in your own schedule, do it at home, do it at the office, or whatever it might be, the results are fast. Yeah. I think the 15-minutes or so, every day, seems to be a pretty easy exchange for a weekly drive to a testing facility.” (PID 22, Adherence: 100%). **Burden 3**: “I usually work-out in the morning, do that, take the test as I was getting ready to jump in the shower. And then, by the time I’m out in the shower, the results are starting to come up. So, it was a pretty easy routine.” (PID 22, Adherence 100) **Burden 4**: “I liked the fact that I didn’t have to put it all the way up my nose like with the other tests, I think that was a big deal that I could just go to one nostril” (PID 13, Adherence: 81%). **Burden 5**: "I do prefer having someone else monitor the situation from a supervisor perspective. I prefer to have someone overseeing the process.” (PID 15, Adherence: 100%) **Burden 6**: Maybe it’s having a two-year-old in the house. But I just could not get in the habit of doing it in the morning…I would often forget to do it to the point where I just started doing it at night. Of the 21 days, there were probably half where I was crawling into bed and said, "Oh my god, I forgot." And then, I had to sit there for 15 minutes when all I wanted to do was go to sleep (PID 20, Adherence 95%)
**Self-Efficacy** An participant’s confidence that they could perform the behavior(s) required to test daily.	**Self-efficacy 1**: “You know, at first when I got the kit, I was like, “Oh, no, I have to learn this thing, I’m not going to be able to do this.” Like, “What do I do?” […] but once I did it once, it was like, “Oh, this is super, super easy.” (PID 43, Adherence 94%) **Self-efficacy 2**: "I think once you do it once or twice, you see is, it’s pretty straightforward. There’s not a lot of ways to screw this one up." (PID 22, Adherence 100%) **Self-efficacy 3**: “I wanted to be sure to do it in the morning so that I didn’t get caught up in my day and forget…I kept [my test kit] on my dining room table to remind me every single morning to do my COVID test…I knew that I needed a certain amount of light in order to photograph with my phone the results…. I just I had all the components out on the table for me so that I could do it as efficiently as possible. And then it became very routine, and it was really easy.” (PID 39, Adherence 95%) **Self-efficacy 4**: “I did sort of the same time every day…Like 1:00 or 2:00 in the afternoon…Because that was the time that I got my first appointment at the PCR. And so, I decided, just try and be regular, and try and do it the same—try and get it in the afternoon every time.”(PID 21, Adherence 76%) **Self-efficacy 5**: “I would prefer to sleep in a little bit on the weekends, so I was having to remember when I got up and not necessarily going straight to my computer that oh, I still need to do that today. And yeah, any day that my schedule varied for some reason, whether it be I needed to go in late that day, or I was up late the night before or something like that that would throw me off. (PID 15, Adherence 100%) **Self-efficacy 6**: “Weekends were the hard ones, right, because you don’t, you don’t have as much routine, or at least your day doesn’t start at the exact same time as it normally does kind of during a business week, but it wasn’t terrible, because you actually have more time as well so it’s not like you’re rushed for anything." (PID 44, Adherence 100%)
**Opportunity Costs** The extent to which benefits, profits, or values must be given up in order to test daily.	**Opportunity cost 1**: I spent just a couple minutes doing the test, but then, you know, the timer of waiting 15 minutes […] and then, you know, I’m on a phone call and now it’s like I had to set a separate alarm to make sure it was five minutes. Now I have to get off the phone call, so I can take a photo. (PID 38, Adherence 100%). **Opportunity cost 2**: I’ve already sort of set aside the time to take the test, but now I have to come back to it and then it expired, because let’s say I was on the phone or something was happening with the kids or whatever and I couldn’t get back in that exact window (PID 38, Adherence 100%). **Opportunity cost 3**: I just had to be really careful about monitoring it. Have the phone nearby like on, on me, because I’m in I usually don’t always have it, especially if I’m like, like cutting papers or something, and my phone was at the machine or something before, then I don’t just didn’t have it on me all the time (PID 32, Adherence 90%) **Opportunity cost 4**: My internet connection at home isn’t so great. So I’d always think I’ll go someplace else to do it. So that’s the only downside of, of the daily test for me was just not having a consistent internet connection at home in order for the, the test to always scan (PID 32, Adherence 90%) **Opportunity cost 5**: “Yeah, that was almost a daily hassle, it almost never would accept my first few tests, for some reason it couldn’t focus on the test itself…I tried like different angles and…it would like focus in for a hot second, and then it would lose focus again…so I would have to scan the cassette multiple times, before it would finally accept one…that was the most frustrating thing that I came across was just my phone did not want to take that photo.“(PID 15, Adherence 100%)
**Ethicality** The extent to which daily testing fit with the participant’s value system	**Ethicality 1**: "I would not be comfortable working with someone in that environment who refused to get a test. So from that perspective, I am more than happy to be tested regularly, to provide comfort to my coworkers that I am okay to be around" (PID 15, Adherence 100%) **Ethicality 2**: "No, I don’t think testing is a personal choice. I think an employer has every right to say, “We’re going to control the environment where we’re working” […] I think, yeah, testing is perfectly ethically fine in my opinion." (PID 43, Adherence 94%) **Ethicality 3**: "I think it’s for the greater good, because there’s more information that we have, the more people we can keep safe" (PID 26, Adherence 95%). **Ethicality 4**: "I kind of feel the same way I feel about seatbelts […] I feel that it’s okay for them to require it. You know, I get that people may not prefer to do it, but I believe that it’s okay for the company to require it, just like I feel it’s okay for you’d be allowed to wear seatbelts." (PID 13, Adherence 81%) **Ethicality 5**: "I mean, I do feel that people should have a choice, but at the same time sometimes people don’t know what’s best for them. So you just have to then tell them what to do. I guess it’s like dealing with a toddler. You just have to tell them what’s best for them, even though they don’t believe it or want to." (PID 13, Adherence 90%) **Ethicality 6**: "We didn’t want to be transmitters, so that was our biggest concern, I was always concerned about getting it somewhere else and bringing it into work by accident […] I know plenty of people […] they went to something as silly as a barbecue or to a church event or something like that, and then they were surprised that they got COVID, and they brought it into work, and now half their team has to isolate and that was the biggest concern for me is that I would have to explain to people that I gave them COVID. And like that for me was an unacceptable thing, and I wasn’t comfortable with that possibility." (PID 15, Adherence 100%) **Ethicality 7**: "I think I look at it, it’s a democratic, it’s a democratic choice. It’s just a really fine line, because it’s just like having HIPAA and privacy, about your healthcare. I think that that’s really important and vital to our freedom" (PID 39, Adherence 95%). **Ethicality 8**: "I think certain things should be required to be shared, like if you are positive of COVID. You know, I think you, you have to inform your employers so they can do the contract tracing, and make sure that no one’s infected. I think there’s a moral obligation around that." (PID 45, Adherence 95%). **Ethicality 9**: "Sometimes, I am shocked by the number of people who are willing to share their lab results with their supervisors, their secretaries and office coordinators…So it makes me very paranoid of what’s happening to my medical information and how free people may be with that." (PID 15, Adherence 100%) **Ethicality 10**: "And then sharing with the public health department, I mean, that makes sense too, because it’s like another entity that needs to keep track of that and they need to keep accurate records of that." (PID 26, Adherence 95%). **Ethicality 11**: "I feel like the CDC […] I don’t trust them right now at all. I think they’re they’ve lost their marbles. […] I don’t believe that they are right to drop the masks yet, I mean we haven’t vaccinated enough people […] So, I don’t trust the CDC I don’t want them knowing anything I’m up to, because they’d be making some bad choices." (PID 25, Adherence 33%) **Ethicality 12**: Well, I would be hesitant to have my results shared in a way that someone would come to my door and say, “You’ve been declared positive, you must now be quarantined in sort of a communistic way… But I would not really want it to be a centralized agency, like the CDC or something, having the right to come in and know someone status,” (PID 21, Adherence 80%) **Ethicality 13**: "We’re in a new world where there’s a lot of connectivity and people’s information is a commodity. So, health information could be compromised in some way." (PID 21, Adherence 80%) **Ethicality 14**: "I just think the more people I don’t want like, institutions knowing, you know, like multiple institutions knowing my health like that […] I don’t know what they’re using that for, if there was more transparency about like, how they’re compiling data, or what exactly they’re tracking, that might be different, but I usually find that there’s seldomly transparency to that degree in these sorts of studies. (PID 25, Adherence 33%) **Ethicality 15**: "I think the functionality of sharing it directly with your provider would be beneficial, because then you’re not having to forward something or like, download or try to get a copy." (PID 26, Adherence 95%)
**Coherence** The extent to which participants understood the purpose of daily testing and how it could result in a safer workplace.	**Coherence 1**: "I think it has amazing applications if there’s a hot zone or spike everyone just does it daily…which is more effective than you know just like a monitoring situation, because again, we’re not in a diagnostic situation, we’re more in a surveillance returning to work route." (PID 45, Adherence 95%) **Coherence 2**: "I think it’s necessary. Absolutely. Particularly in this business, where individual services are so key to the value of the product." (PID 21, Adherence 80%) **Coherence 3**: "You know, and in the early days […] people are afraid to even go outside. It, it is a kind of a nice safety net to have this type of thing. And I do think the applications are, if we have some hot spot come up with a variant, and you know, it’s an instant solution for you." (PID 14, Adherence 100%) **Coherence 4**: "My sister and her daughter … got COVID and brought it home…if she had had like, you know, that testing on a daily basis available to her, she probably would have known sooner you know, before for that." (PID 32, Adherence 90%) **Coherence 5**: "The bigger issue for us in the workplace is how transmissible it is, once you’re vaccinated. So, I think once that data gets a little more widely researched, then that’ll define the testing a bit more. For now, we haven’t changed testing protocols based on vaccination." (PID 22, Adherence 100%) **Coherence 6**: "Like if the CDC were to recommend and say, “Hey, you know, COVID-19 is no longer a problem we have such low numbers, we recommend that testing is no longer needed,” then I would probably go along with that." (PID 13, Adherence 100%) **Coherence 7**: "Out of the 30,000 tests that we do a week we get an average of seven to eight positives, worldwide. That’s a pretty expensive proposition to find eight people out of 30,000 tests." (PID 4, Adherence 100%) **Coherence 8**: "We are very rapidly heading to a situation where testing at the frequencies we do may not be necessary, but it’s not going to be soon […] I do see there being less purpose for it, I could see it going away eventually for vaccinated individuals. But we would have to be like way past the risk level that we’re at, even though it’s very low." (PID 15, Adherence 100%)
**Perceived effectiveness** The extent to which participant’s believed testing daily would achieve its purpose of keeping the workplace safe	**Perceived effectiveness 1**: "First of all the company I work for who I trust was engaged in this test, number one, number two, it was the—the source of the testing at Columbia. Number three, the absolute detail was reassuring to me every bit of it, because I am very detail oriented, and that’s what I do for a living is requires a lot of detail. And so I was, I found that to be very again, very satisfying, so it gave me a lot of confidence in the quality of the test." (Adherence: 95%) **Perceived effectiveness 2**: "I trust scientists. I trust people who are smarter than me. I trust experts, I guess. That’s always been our MO [modus operandi]. […] And if an expert says, "Next month, as long as you take this every day and get a negative, you’re good to go to work," then I’ll go to work. So, that’s how I think it could affect my behavior now that we’re coming out of that stretch, where it didn’t really matter what you were testing, you still needed to be really locked up." (Adherence 95%) **Perceived effectiveness 3**: ‴I trusted them as much as I would trust any home pregnancy test […] I didn’t have any reason to feel that there was something less trustworthy about them than any other test." (Adherence: 94%) **Perceived effectiveness 4**: “Having it daily like that is nice because I know, I only took the last test 24 hours ago and I’m still testing negative, so the timing is comforting, but I don’t like to give people the false sense of security that they’re still okay, which was a challenge with PCR testing, because you take the test 24 to 48 hours ago, and you just now got your negative result, so I must be okay right now, which of course two days is past so that can be false, like you may have developed it or incubated enough in that time period." (Adherence: 100%) **Perceived effectiveness 5**: "There’s nothing that made me feel worried I just felt that the only thing I picked up on is that these aren’t as accurate as PCR tests. So, you know, the question that I would always kind of get is that those aren’t, just aren’t that accurate, you know so. […] Those are the questions that I would get from friends and family or even, you know, people in the medical field when I would tell them that I was taking these tests." (Adherence 100%) **Perceived effectiveness 6**: "I don’t worry about myself, because I know that I’m going to get a good sample, I don’t know about everyone that always worries me because we do have some people who goes through our test sites, when I’ve watched them, their noses are really dry or they’re nervous, so they won’t really get a good sample." (PID 13, Adherence 81%) **Perceived effectiveness 7**: "I think there is something for some people, the idea of a doctor or a nurse or some professional doing the test, versus you doing it yourself, there’s going to be some expectation that, the one may be more reliable than the other." (PID 22, Adherence 100%) **Perceived effectiveness 8**: "We’re depending on people to do this themselves, to keep people honest that they’re doing it, and that they’re not finding a workaround for the system, versus the current process, where they’re being tested by a clinician, and results are coming to us. So, that I think is the only part that would have to get sorted out, is exactly how we guarantee that they’re doing the test themselves." (PID 22, Adherence 100%) **Perceived effectiveness 9**: "People start to use the psychology of, but I get tested so yeah, I went to the bar last night and I hung out with my friends, but don’t worry I get tested, and you’re like, the test didn’t do anything for you, you understand that? [They will say things like] "I’m getting tested, and I just want to live my life" (PID 44, Adherence 100%) **Perceived effectiveness 10**: "The more testing we do […] the more—the more frivolous people come in their normal day-to-day life, because they say it’s okay, I’m getting tested so it’s fine. Instead of testing being the result of you doing everything else right." (PID 44, Adherence 100%)

For some participants who struggled to incorporate testing into their daily routine or who had difficulty using the mobile app to capture and interpret their test result, daily testing was more burdensome. In these cases, participants reported feeling dread when they remembered they still had to test or frustration at being repeatedly unable to scan in a test result through the mobile app (Burden 6, Opportunity cost 5).

#### Burden

More than half of participants disagreed with the statements “it is inconvenient to use the self- test every day” (61%) (Table 3). Most participants describing daily self-testing as very convenient compared to other testing options they experienced (e.g., large drive-through testing events early in March/April 2020 or mandatory testing offered by their employer). Even among participants who had access to weekly on-site testing at their workplace, most preferred the daily antigen test. These participants overwhelmingly described testing daily as fast and easy often noting that daily self-testing took less time overall than weekly PCR testing (Table 4, Burden 1); that they didn’t mind daily testing because they could test on their own schedule and get the results quickly (Burden 2) or incorporate self-testing into other established routines (e.g., timing the 15-minute waiting period with a shower) (Burden 3); and that they did not find the daily swabs to be invasive (Burden 4). While some participants described mild discomfort from the daily swabbing, this was not a major burden.

In the minority were participants who acknowledged that while daily at-home antigen testing was more convenient than weekly PCR testing, they still preferred testing using PCR because of the mental energy required to self-administer the test (Burden 5). Other participants described difficulty incorporating testing into their daily routine and reported testing fatigue (Burden 6).

#### Self-efficacy

Participants reported that the self-test was easy to learn to use (90%) and results easy to understand (100%) (Table 3). However, only half of participants (55%) stated that they developed a habit of testing for COVID-19 in their daily routine, with experiences adhering to daily testing varying widely across participants. While some participants reported feeling initially overwhelmed by the testing steps, nearly all reported mastering the steps after a few days of testing (Table 4, Self-efficacy 1, 2). Factors that supported daily adherence included developing strategies to streamline the testing process (Self-efficacy 2); incorporating the test into their daily routine (Self-efficacy 3, 4); and placing the test kit in a prominent location (Self-efficacy 3). In contrast, participants reported that changes to their daily routine and weekends were the greatest barrier to daily adherence (Self-efficacy 5, 6).

#### Opportunity cost

Most participants strongly agreed with the statement: “It is easy for me to find a time to use the test every day” (68%) (Table 3). Nonetheless, time was often described as the biggest opportunity cost to testing daily (Table 4, Opportunity cost 1, 2). Some participants attempted to minimize the time cost by multi-tasking (Burden 3) or spreading out the time commitment by setting up their test kit the night before (Self-efficacy 3). However, others reported that efforts to multi-task resulted in more time spent testing because they would lead to mistakes like missing the results window during and having to repeat the testing process again (Opportunity cost 1, 2, 3). For other participants, access to strong internet and/or a good light source were additional opportunity costs to daily testing as the mobile app required a specific level of light to accurately interpret the test result (Opportunity cost 4, 5). Participants who struggled to upload a picture of their test result consequently described testing as a “daily hassle” and “frustrating”, negatively impacting their affective attitude about the testing regimen.

#### Ethicality

Most participants agreed that it is ethical for an employer to require its employees to test regularly for COVID-19 (87%) (Table 3). However, a small minority believed that regular testing should be a personal choice (10%) and a significant portion of participants believe that sharing test results should be a personal choice (42%). Many participants articulated how working long hours near other coworkers necessitated the need for routine testing and saw mandatory testing as a practical approach to keeping the workplace safe (Table 4, Ethicality 1, 2). Specifically, many used Utilitarian arguments to justify mandatory testing, saying that the small loss of privacy and autonomy was justified for “the greater good” of workplace safety (Ethicality 3, 4, 5). Others went further, stating that routine testing helped them fulfil their personal responsibility to keep their workplace safe, noting that they “didn’t want to be transmitters” and that giving someone else COVID would be “an unacceptable thing” (Ethicality 6). In contrast, a small number of participants believed mandatory workplace testing was not ethical as it infringed upon individual liberties. While these participants were personally willing to test regularly through their employer, they believed that coercive tactics should not be used to keep the workplace safe (Ethicality 7).

While participants tended to feel strongly that requiring regular COVID-19 tests was either ethical or unethical, participants’ feelings about who should have access to test results and what this health information should be used for was more nuanced. Many agreed that test results should be reported to their employer to prevent the spread of infection should an employee test positive (Ethicality 8); however, some had concerns about who within their company should have access to results (Ethicality 9). Participants also had mixed feelings regarding whether COVID-19 test results should be shared with larger health organizations, such as state health departments or the Centers for Disease Control and Prevention (CDC). Most participants believed these were trusted entities and saw the benefit of sharing COVID-19 results for contract tracing, surveillance, and allocation of health services (Ethicality 10); however, this feeling was not universal, and some reported not trusting the CDC or worrying that the health department would limit their freedom if they tested positive (Ethicality 11, 12). Additionally, a small number of participants believed test results should stay within their employer due to concerns about health information privacy, including fears that their health information could be sold or compromised (Ethicality 13, 14). At the same time, many participants liked the idea of their test results being automatically reported to their employer or doctor through a mobile app, as this would eliminate the need for a third party to handle test results (Ethicality 15).

#### Coherence

The majority of participants believed that testing for COVID-19 daily made sense to prevent the spread of COVID-19 at and beyond the workplace (77%) (Table 3). Support for daily testing was particularly strong for times when COVID-19 rates were increasing or high; in this context, regular testing was considered an effective and necessary strategy for monitoring infection rates and preventing COVID-19 transmission (Table 4, Coherence 1). Specifically, many participants believed the unique needs of the entertainment industry, which relies on non-replaceable people to fill specific roles, further necessitated a stricter approach to COVID-19 control to ensure that work would not be disrupted (Coherence 2). Many participants said they wish they had access to daily testing during the original peak of the pandemic in March and April of 2020 (Coherence 3).

While support for regular testing was near universal during times when risk of COVID-19 infection was perceived to be high (i.e., before vaccines were available, when rates of infection were still increasing), there was less agreement about when routine testing was no longer needed. Many participants thought it made sense to continue regular COVID-19 testing until there was more research available on the transmissibility of emerging COVID-19 variants and effectiveness of the COVID-19 vaccines (Coherence 5). Others preferred to listen to scientific recommendations from public health agencies to decide whether frequent testing made sense (Coherence 6). Some participants expressed increasing doubt that regular COVID-19 testing was still necessary given that vaccines were widely available to all adults and rates of COVID-19 in the workplace and surrounding community were very low (Fig 1). One participant in a managerial position used a cost benefit analysis to argue that frequent testing did not make sense from a financial perspective given the low positivity rates (Coherence 7). Many believed testing did not make sense for vaccinated populations, but that it may be an alternative for those who did not wish to become vaccinated (Coherence 8).

#### Perceived effectiveness

Participants had high confidence in the effectiveness of a daily testing intervention with the majority reporting that they trusted the results of the at-home antigen test (81%) and nearly all expressing confidence that testing daily will keep the people they work with safe from COVID-19 (90%) (Table 3). Whether participants perceived the daily testing intervention would keep the workplace safe depended on three factors: 1) whether they trusted the test results, 2) whether they believed their co-workers would be willing and able to test daily, and 3) whether they believed frequent testing would introduce any unintended consequences. Although the antigen test used in the study had not yet received FDA approval and was only for use in research settings, nearly all participants reported trusting the results, citing trust in Columbia University (which helped develop the test in collaboration with a biotechnology company), trust in Disney (their employer) to provide a safe and effective test, and trust in experts/scientists (Table 4, Perceived effectiveness 1, 2). Other participants drew on previous experience using similar at-home tests (e.g., home pregnancy tests, glucose monitoring, maintaining swimming pool pH) as reasons for trusting the results (Perceived effectiveness 3). One participant noted that they trusted the daily antigen test result more than weekly PCR because the antigen test result reflected current COVID-19 status while the PCR result reflected a status from 24–48 hours ago and could give a false sense of security (Perceived effectiveness 4). In contrast, a different participant reported trusting the antigen test result less than PCR because they had heard from family and friends with medical training that antigen tests were not as accurate as PCR (Perceived effectiveness 5).

While participants were generally confident in their own ability to successfully use the at-home antigen test, they were less confident about their co-workers’ abilities, which they believed could undermine the effectiveness of the daily testing intervention. These concerns included not believing that their coworkers would obtain a sufficient swab sample and properly self-administer the test at home (Perceived effectiveness 6), believing that a test administered by a medical professional would be more accurate (Perceived effectiveness 7), and worry that the identity of the test taker and the test result could not be confirmed in a home setting (Perceived effectiveness 8). Finally, some participants raised concerns that a frequent testing regimen would lead to employees increasing risky behavior that could result in more COVID-19 transmission (Opportunity cost 9, 10).

## Discussion

As the COVID-19 pandemic continues to evolve with the emergence of new variants [18,19], waning vaccine and natural immunity [20–22], advancements and setbacks in treatment options [23], and changing human behavior in response to these inputs [24,25], new strategies for controlling and adapting to COVID-19 are needed. The Delta and Omicron variants have brought heightened interest in at-home antigen self-testing and the Biden administration has responded with significant resources to rapidly increase the availability of antigen tests in the US beginning late January 2022. The extent to which this intervention will have a significant public health impact remains to be seen but will in part depend on whether, and if so, how, Americans will use these tests. However as of January 2022, to our knowledge no studies have examined the acceptability of self-testing for COVID-19, including serial testing regimens that have been shown most effective at detecting cases.

Our mixed-methods study on the acceptability of a serial testing regimen showed that daily testing using an at-home antigen COVID-19 test was acceptable in one employment context. Employees were willing and able to adhere to a daily testing regimen for up to 21 days, with 75% of participants achieving adherence 90% or greater. In addition to reporting overall high acceptability and adherence, our study identified three key implementation challenges that must be addressed for antigen tests to reach their full public health potential.

First, there is a significant need for educational campaigns to build lay user trust in the accuracy of at-home antigen tests and knowledge around when and how to use them, including how to act on test results [26]. Of the seven constructs that comprise acceptability, coherence and perceived effectiveness were most salient and determined whether participants ultimately felt it “was worth it” to test for COVID-19 daily. Importantly with respect to coherence, participants expressed a willingness to tolerate burden (inconvenience, invasiveness), opportunity costs (time), threats to ethicality (loss of privacy) associated with daily testing when they perceived the threat of COVID-19 to be high (high coherence), but indicated that given testing fatigue, this tolerance would not last forever. In the face of declining cases and universal access to vaccination among adults, participants saw a time in the then near future when this testing frequency would no longer be necessary, rendering the intervention less acceptable. As shown in Fig 1, the study was implemented right before the emergence of the Delta variant when case numbers were decreasing, vaccination eligibility was extended to study participants, and the fear of COVID was palpably receding in the public domain and among our study participants. Respondents reported that daily at-home testing made less sense at this moment in the epidemic than it had one month earlier when case numbers were rising and vaccinations were not yet approved. These findings highlight that people’s tolerance for testing intensities change based on their changing perceptions of risk. Users urgently need clear public health guidance around when and how frequently to use at-home antigen tests under different epidemiological conditions, including specific guidance around on- and off-ramps to different testing intensities [27].

We also found that trust in the accuracy of the at-home antigen test was key to whether participants perceived that the intervention would be effective. While most participants reported trusting the results of the test, this trust was primarily grounded in trust in the institutions providing this test and not in scientific evidence supporting test accuracy. Yet given that antigen tests will be distributed and used outside of the employment context, educational campaigns are needed to build trust in the tests, including by increasing lay user knowledge about the accuracy of antigen tests for detecting when users are most infectious and able to transmit to others. Increasing user literacy around antigen testing for COVID-19 may be particularly important given emerging mixed evidence about the performance and accuracy of antigen tests with the Omicron variant, including concerns about tests being less sensitive [28–31] and slower to detect COVID-19 in the nose compared to other sites [32], which may erode trust in antigen tests and impact acceptability and adherence to testing regimens. This work will need to be ongoing and responsive to emerging information with each new variant and delivered across all communities to ensure that scale up antigen testing does not exacerbate existing health disparities [7,33].

Second, we wish to highlight that any frequent testing campaign will generate an enormous amount of sensitive health data and there is currently no existing universal infrastructure for routinely capturing or reporting this information to relevant health authorities, health care providers, or institutions (e.g., employers, schools). The lack of a universal recording and reporting system for at-home antigen test results that are test brand agnostic creates numerous challenges including 1) potentially biased statistics around COVID-19 infection rates due to millions of home antigen test results not being reported, with implications for public health surveillance and resource allocation [34–36]; 2) sharing of COVID-19 test results through non-secure channels (e.g., text messages, emails) providing opportunities for privacy breaches [37–39]; and 3) challenges for the delivery of anti-viral treatments for COVID-19 within three days after symptom-onset for maximum benefit, due to the lack of guidance on whether a positive home antigen test can be used to qualify for treatment [40,41]. In our study, testing data was captured securely and systematically using a mobile app that recorded and interpreted the test result for the user; these data were only shared with the study team, but the app could be further developed to support secure sharing with other entities. Most participants in our study found the app to be acceptable, however using this app was not without challenges, and some participants experienced significant difficulty using the app daily due to lack of internet or smart phone operating system too old to support the app. In a less technology savvy population, comfort with smart phone technology may also be a significant barrier. Further, while nearly all participants were comfortable with tests results being shared with the research team and their employer, they expressed significant concern about results being shared with health or government entities aside from their own provider.

Anticipating potentially more dangerous pandemics in the near future, the Biden administration has developed a five-pillar plan for pandemic preparedness that includes “transforming our medical defense [through] dramatically improving diagnostics” (Pillar 1) and “ensuring situational awareness about infectious disease threats for both early warning and real-time monitoring” (Pillar 2) [42]. A connected at-home antigen test that could accurately capture tests results and share them with government and health authorities in real time would address both pillars, however significant work remains to be done to build public trust and acceptability in such a system [43]. Within the current pandemic, anecdotally, antigen test results are already being informally shared with institutions like schools and employers to support strategies like “test to stay” (testing to remain after exposure) and “test to return” (testing to end isolation or return after infection). To our knowledge, there has been little to no research done on how these results are currently being shared or stored; guidelines for best practices are urgently needed.

Finally third, given that the COVID-19 and future pandemics may disproportionally and negatively affect low-income and marginalized communities, it is essential that serial testing interventions in the workplace and elsewhere be employed in ways that put equity at the forefront. Mandatory workplace testing policies have the potential to both protect the most vulnerable, front-line employees, but if instituted without proper protections, can also create negative unintended consequences that may disproportionately burden vulnerable populations. The acceptability of a daily testing regimen in this study population was likely influenced by the employment context, including already established norms around routine COVID-19 testing, and job security and benefits (e.g., health insurance, dedicated paid COVID-19 sick leave) available to participants in our study who were all employed full-time. Although we anticipated and probed for worries about job security or loss of income due to testing positive for COVID-19, hypothesizing that this could reduce acceptability and adherence to the intervention, no participants reported such concerns. Possibly because of their job security, participants also almost universally believed that mandatory COVID-19 testing in the workplace was ethical. In other employment contexts with less job and income protection, mandatory serial testing may be much less acceptable and could have harmful unintended consequences.

## Conclusion

Our study contributed to the knowledge gap around the implementation of serial at-home rapid antigen testing regimens. This is the first study to our knowledge to evaluate the acceptability of a serial COVID-19 testing regimen with a connected test in an employment context and report on adherence to the intervention. Our study advanced the Theoretical Framework of Acceptability by developing quantitative measures of acceptability and identifying three critical barriers to the successful implementation of rapid antigen testing as a public health intervention.

There were also a number of limitations. The study was implemented at a time of relatively low COVID worry as the number of cases was falling steeply and vaccines offered a promise of durable protection. This epidemiological context along with the COVID-19 vaccine exclusion criterion impacted our ability to enroll a larger number of study participants and the relatively small sample size limits the generalizability of the findings and our ability to reach saturation. Therefore, results should be interpreted with the small sample in mind. However, low accrual highlighted that context is an understudied component of acceptability causing acceptability to vary across time. Generalizability may also be limited by the fact that the small study was conducted in Southern California in a politically liberal area with high support for COVID precautions and within a stable employment context. Participants were all fully employed at a large company (Disney), and race, education, and SES are not representative of the broader US population. Thus, findings may not be generalizable to other contexts, including other countries or epidemiological contexts, or populations. The relatively high adherence to the intervention we report is likely an artifact of the study context and selection bias based on willingness to enroll in the parent daily testing study and participate in in-depth interviews about the experience; we would expect adherence to be lower in the real world. Finally, we only looked at serial testing in an employment setting, although serial testing has now been rolled out in other contexts. Additional studies are needed to build the evidence-base about the acceptability of and adherence to serial-testing regimens to evaluate their public health contribution to interrupting transmission.

## Supporting information

S1 Fig To test for COVID-19, participants completed the following steps show in in the instructions below: (1) swabbed anterior nares of each nostril (i.e., shallow swab); (2) placed the swab in a plastic tube and added drops of buffer solution; (3) swirled the swab inside the tube with the buffer solution and squeezed the tube to mix the buffer solution with the swab; (4) discarded the swab and placed a dropper cap on the tube containing the buffer solution; (5) squeezed drops of the buffer solution onto a test cassette (6) waited 15 minutes to read the test result (1 line = negative, 2 lines = positive).
(DOCX)
