# Supplementary material for: A mixed methods study evaluating acceptability of a daily COVID-19 testing regimen with a mobile-app connected, at-home, rapid antigen test: Implications for current and future pandemics
Source: PLoS One. 2022 Aug 8;17(8):e0267766. doi: 10.1371/journal.pone.0267766 (PMC9359568; doi:10.1371/journal.pone.0267766)
Supplement: S1 Fig — To test for COVID-19, participants completed the following steps show in in the instructions below: (1) swabbed anterior nares of each nostril (i.e., shallow swab); (2) placed the swab in a plastic tube and added drops of buffer solution; (3) swirled the swab inside the tube with the buffer solution and squeezed the tube to mix the buffer solution with the swab; (4) discarded the swab and placed a dropper cap on the tube containing the buffer solution; (5) squeezed drops of the buffer solution onto a test cassette (6) waited 15 minutes to read the test result (1 line = negative, 2 lines = positive). (DOCX) [file pone.0267766.s001.docx]

**Supporting Information**

**Figure 1. At-Home COVID-19 Antigen Test Instructions Delivered via Mobile Application**

To test for COVID-19, participants completed the following steps showin in the instructions below: (1) swabbed anterior nares of each nostril (i.e., shallow swab); (2) placed the swab in a plastic tube and added drops of buffer solution; (3) swirled the swab inside the tube with the buffer solution and squeezed the tube to mix the buffer solution with the swab; (4) discarded the swab and placed a dropper cap on the tube containing the buffer solution; (5) squeezed drops of the buffer solution onto a test cassette (6) waited 15 minutes to read the test result (1 line = negative, 2 lines = positive).

| 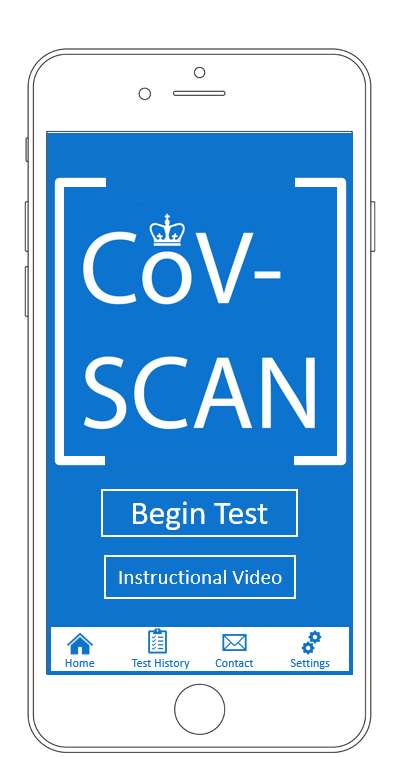 | 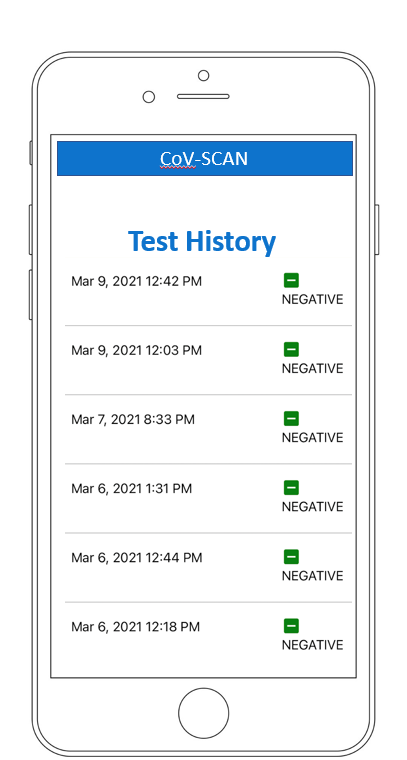 | 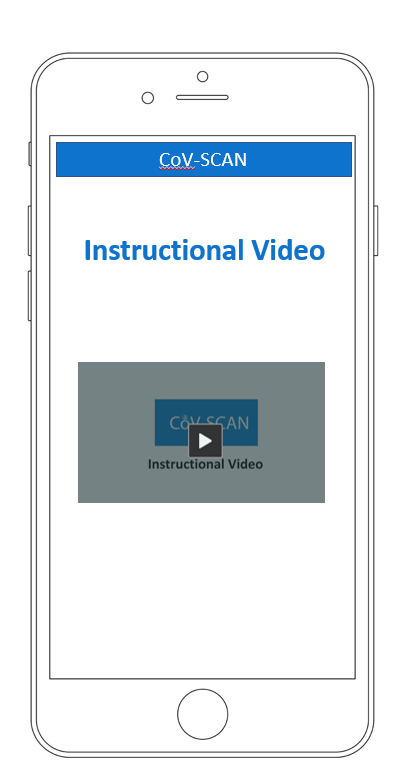 |
| --- | --- | --- |
| 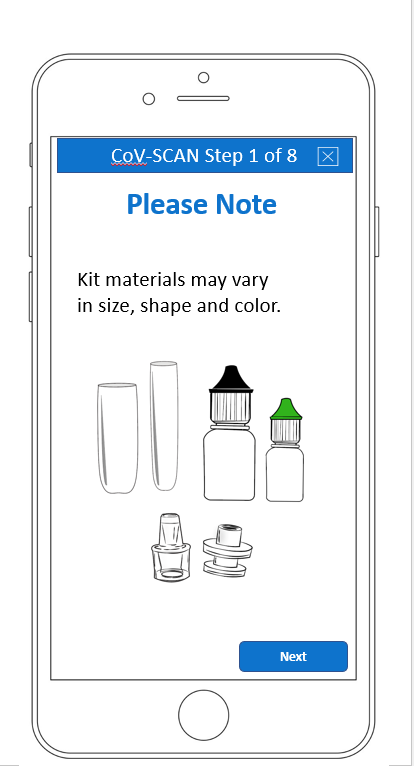 | 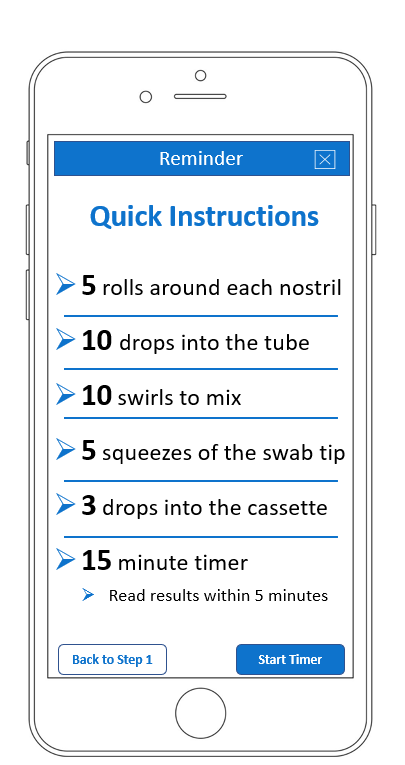 | 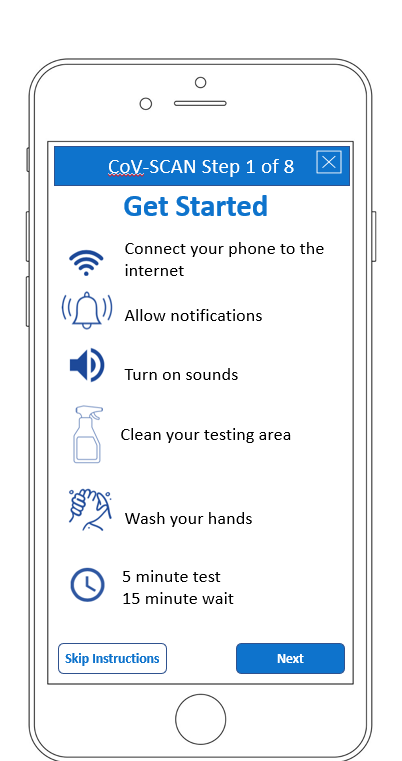 |
| 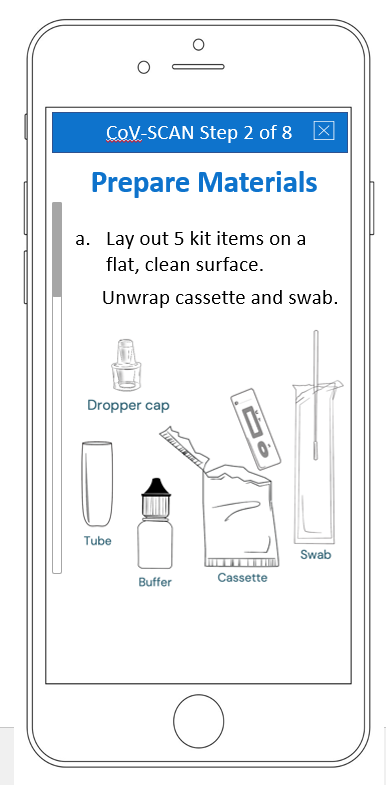 | 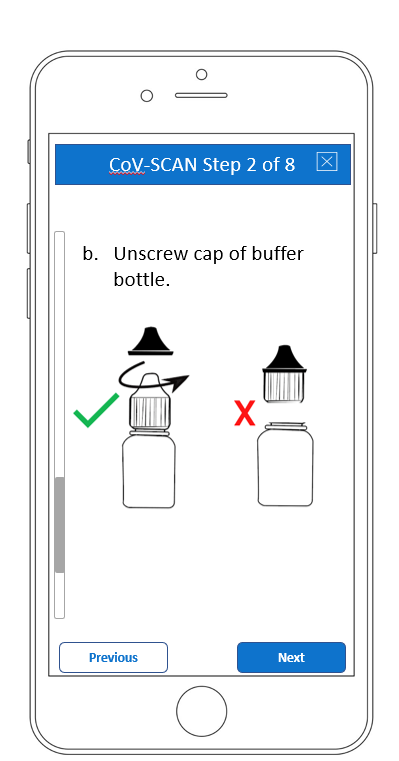 | 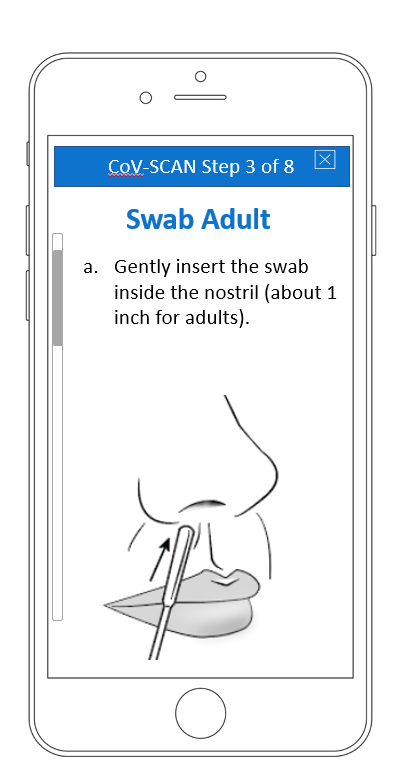 |
| 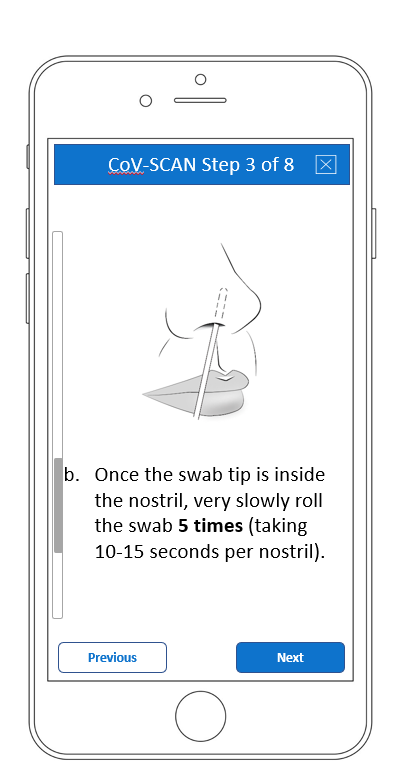 | 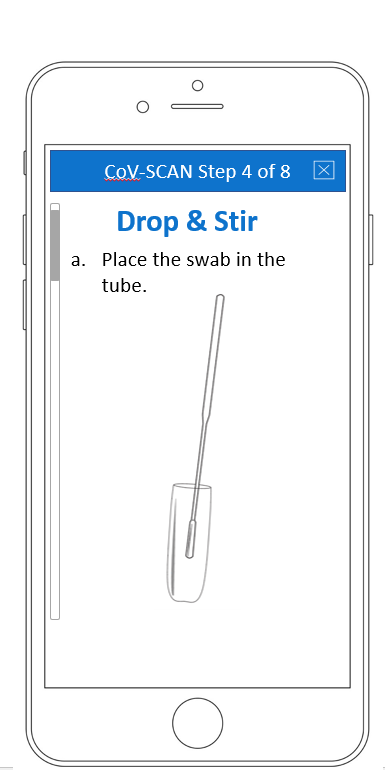 | 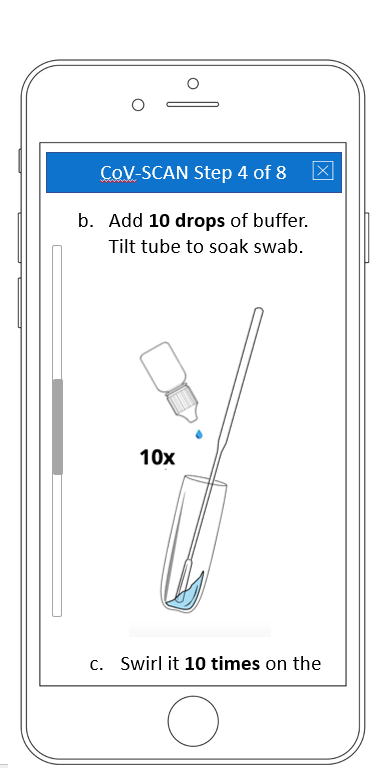 |
| 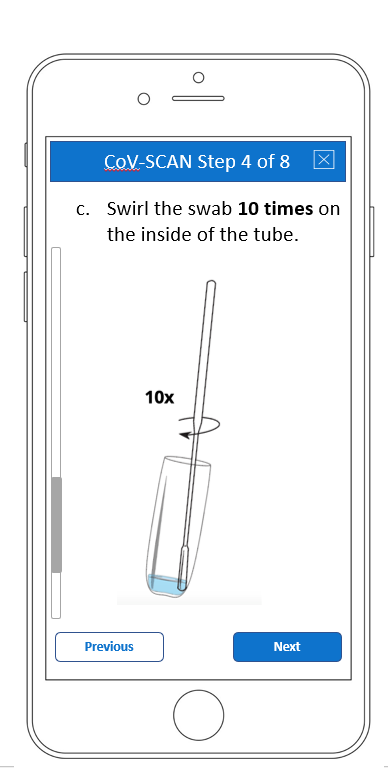 | 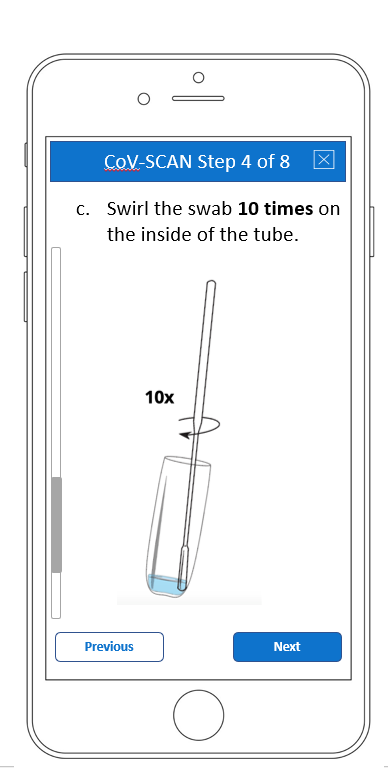 | 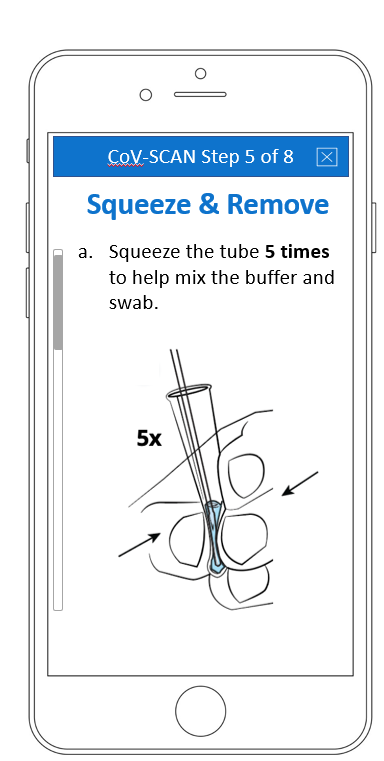 |
| 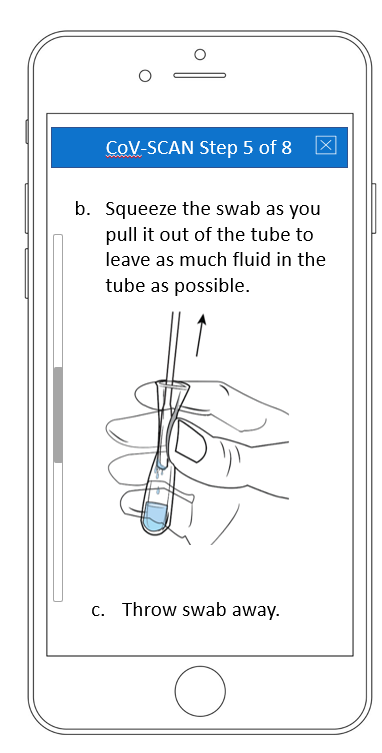 | 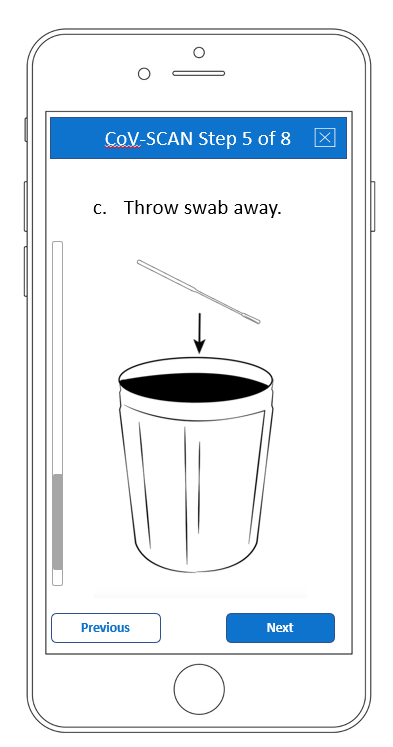 | 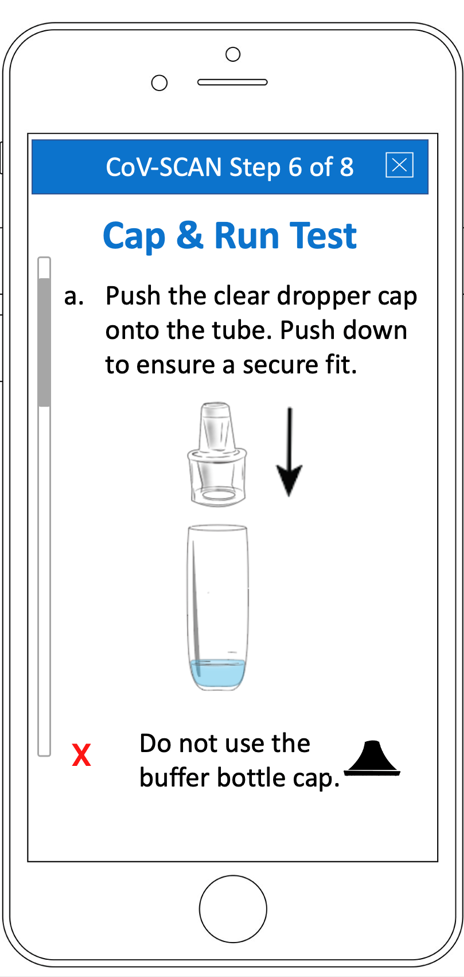 |
| 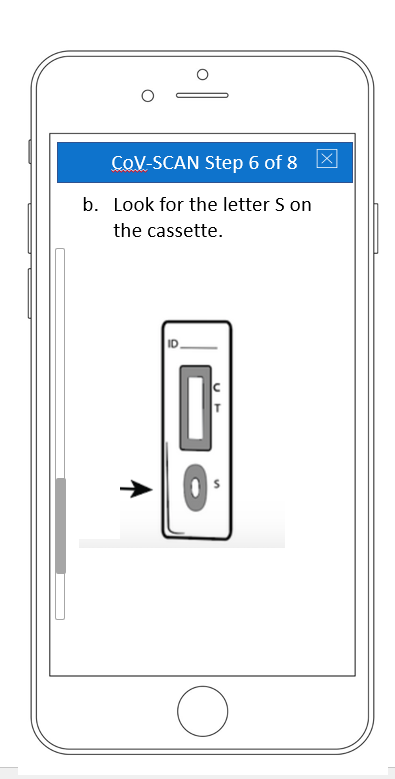 | 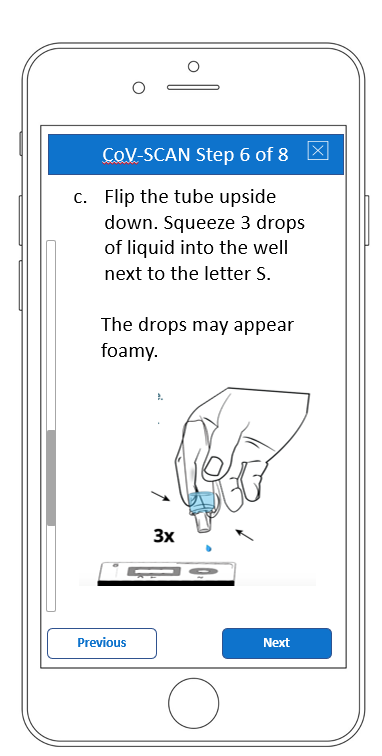 | 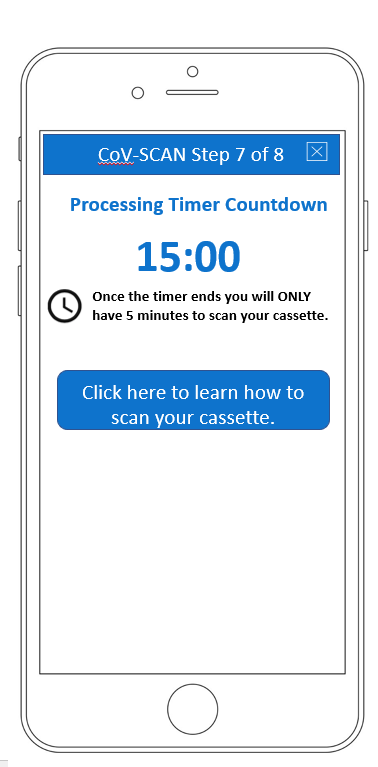 |
| 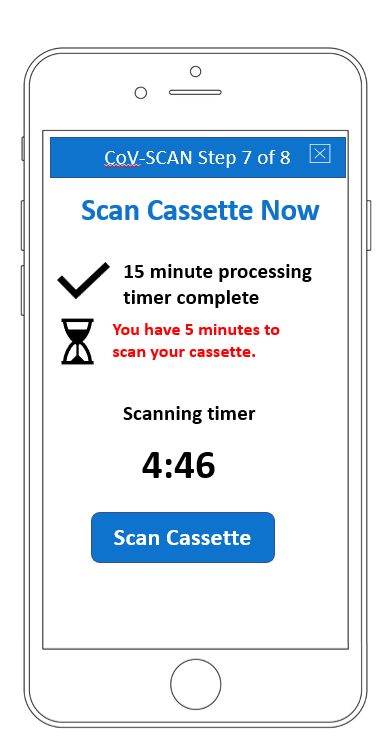 | 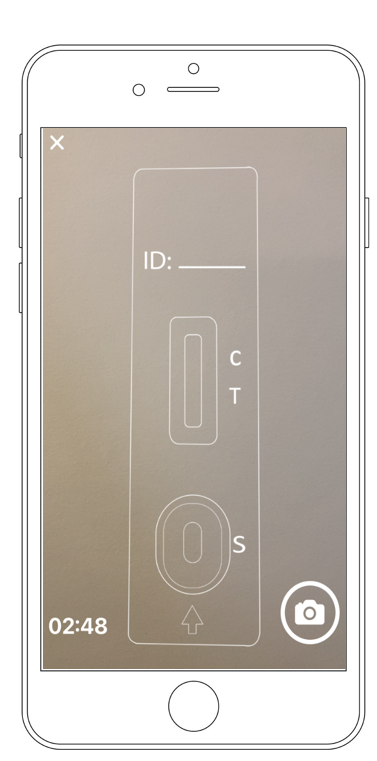 | 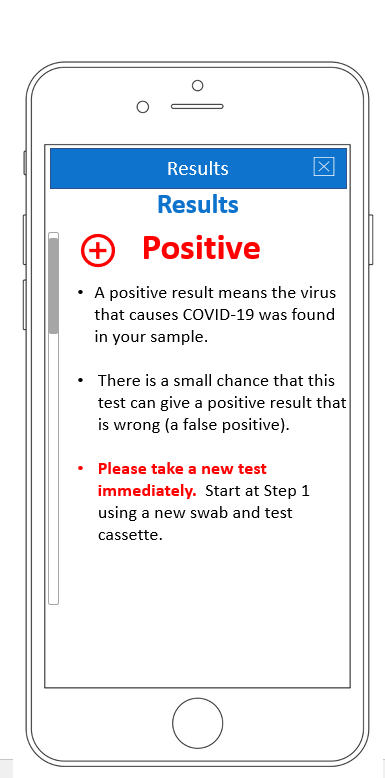 |
| 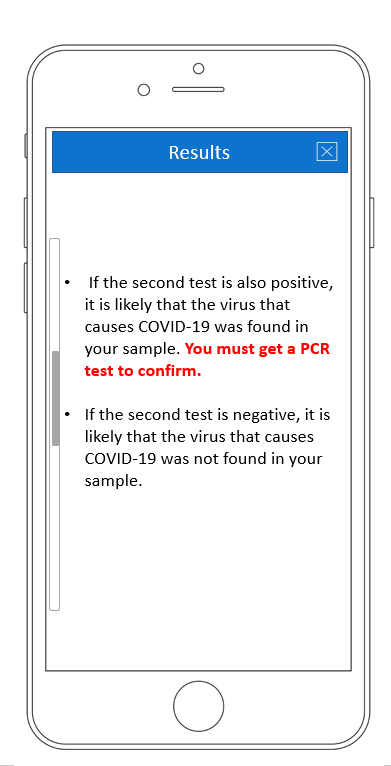 | 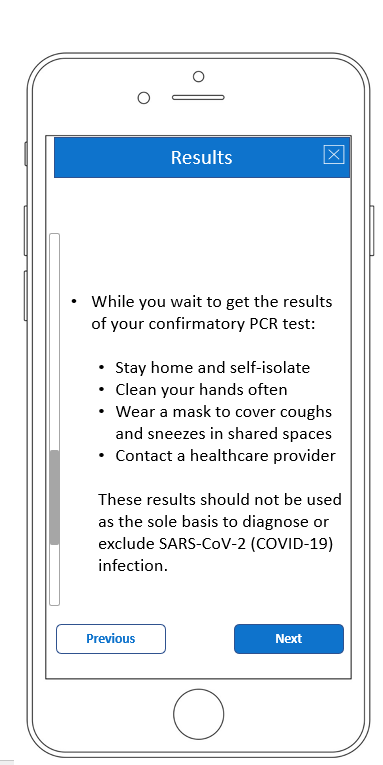 | 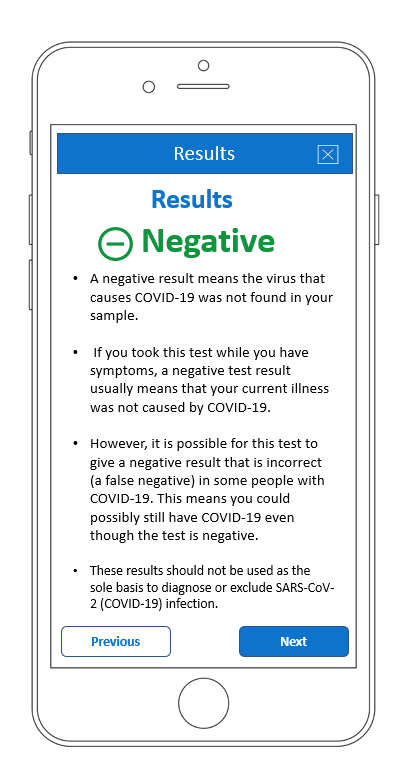 |
| 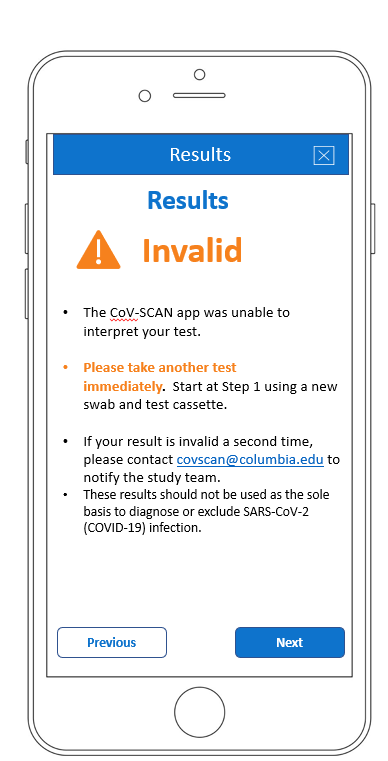 | 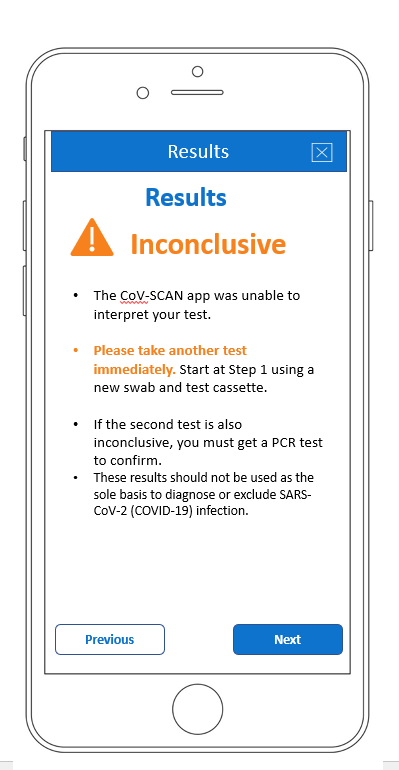 | 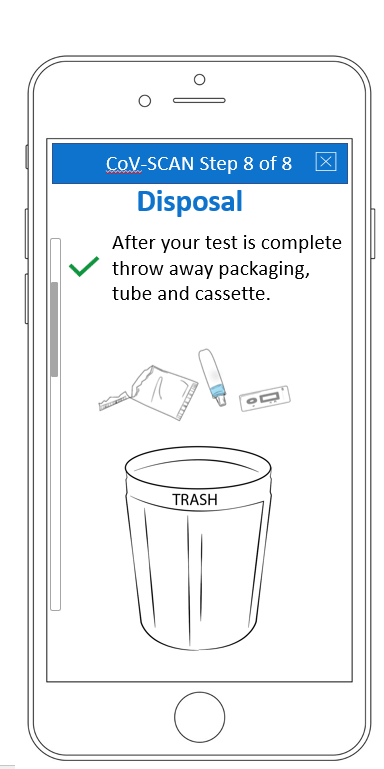 |
| 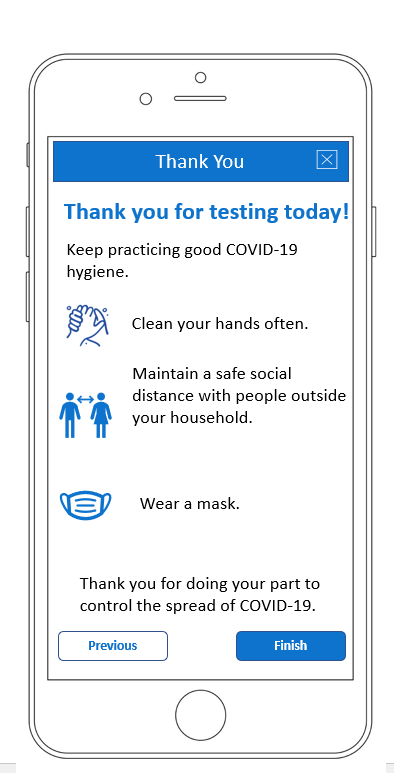 |  |  |
